# Complex harmonic regularization with differential evolution in a memetic framework for biomarker selection

**DOI:** 10.1371/journal.pone.0210786

**Published:** 2019-02-14

**Authors:** Sai Wang, Hai-Wei Shen, Hua Chai, Yong Liang

**Affiliations:** 1 Faculty of Information Technology, Macau University of Science and Technology, Taipa, Macau; 2 State Key Laboratory of Quality Research in Chinese Medicines, Macau University of Science and Technology, Taipa, Macau; Fred Hutchinson Cancer Research Center, UNITED STATES

## Abstract

For studying cancer and genetic diseases, the issue of identifying high correlation genes from high-dimensional data is an important problem. It is a great challenge to select relevant biomarkers from gene expression data that contains some important correlation structures, and some of the genes can be divided into different groups with a common biological function, chromosomal location or regulation. In this paper, we propose a penalized accelerated failure time model CHR-DE using a non-convex regularization (local search) with differential evolution (global search) in a wrapper-embedded memetic framework. The complex harmonic regularization (CHR) can approximate to the combination $\ell_{p}\;\left(\frac{1}{2}\leq p<1\right)$ and *ℓ*_*q*_ (1 ≤ *q* < 2) for selecting biomarkers in group. And differential evolution (DE) is utilized to globally optimize the CHR’s hyperparameters, which make CHR-DE achieve strong capability of selecting groups of genes in high-dimensional biological data. We also developed an efficient path seeking algorithm to optimize this penalized model. The proposed method is evaluated on synthetic and three gene expression datasets: breast cancer, hepatocellular carcinoma and colorectal cancer. The experimental results demonstrate that CHR-DE is a more effective tool for feature selection and learning prediction.

## 1 Introduction

Feature selection is a great step forward for selecting biomarkers in biological data with high dimension and small sample. Among various kinds of feature selection methods, the regularization methods use different penalty functions embedded in the learning procedure into a single process and has lower risk to over-fitting. The well known penalty is the least absolute shrinkage and selection operator (Lasso, *ℓ*_1_-norm) [1], which is performing continuous shrinkage and feature selection at the same time. Other *ℓ*_1_-norm type regularization methods typically include smoothly clipped absolute deviation (SCAD) [2], group lasso [3], minimax concave penalty (MCP) [4], etc. Besides, Xu et al [5] has proved that when $0<p<\frac{1}{2}$, there is no significant difference in the performance of *ℓ*_*p*_-norm, but the computational complexity to solve the *ℓ*_1/2_ regularization is much lower than that of the *ℓ*_0_-norm; while $\frac{1}{2}<p<1$, the solutions of the *ℓ*_*p*_ regularization is more sparse with the decline in *p*. Under this theory, Chu et al [6] proposed a naïve harmonic regularization that can approximate $\ell_{p}\;\left(\frac{1}{2}\leq p<1\right)$ penalties.

One limitation of these *ℓ*_1_-norm type regularizations is that when the data set contains strong correlations among the predictors, it tends to select only one feature from the group and does not even care which one is selected, but these groups may be gene pathways in gene expression data. In theory, a strictly convex penalty function provides a sufficient condition for grouping effect of variables and *ℓ*_*q*_-norm (*q* > 1) penalty guarantees strict convexity [7]. Zou and Hastie [8] proposed the Elastic net that mixes the *ℓ*_1_ and *ℓ*_2_ penalties. After that, some regularization methods without prior knowledge that combined *ℓ*_2_-norm for selecting groups of variables are SCAD-*ℓ*_2_ [7], *ℓ*_1/2_ + *ℓ*_2_ [9], and so on. While, there are also some regularization methods with prior knowledge, such as group lasso [3] that has been used for multivariate analysis of variance model, where each factor may have several levels and can be expressed by a group of dummy variables. In this article, we employ a complex harmonic regularization (CHR) [10] that approximates to the combination $\ell_{p}\;\left(\frac{1}{2}\leq p<1\right)$ and *ℓ*_*q*_ (1 ≤ *q* < 2) to select the key factors in group among all features. This approach avoided determining the value of *p* or *q* in advance, i.e., we would not need to assume the probability distribution of the data, before evaluating the grouping effect and spare by the existing regularization methods.

However, the hyperparameters of CHR are sensitive to the resolution, and the hyperparameter tuning is typically done by expert analysis, evolutionary algorithms, bayesian optimization and grid search [11]. Jaderberg et al [12] efficiently set the hyperparameters of neural networks based on the genetic algorithm (GA). Liu et al [13] proposed a hybrid genetic algorithm which combines genetic algorithm with embedded *ℓ*_1/2_ + *ℓ*_2_ regularization together. Such evolutionary algorithms are suitable to deal with tuning hyperparameters of these multimodal penalty functions. GA [14] is the most widely used one in the literature. However, GA is much slower convergence to optimum for high dimensional problem. Consequently, it cannot handle the learning model with more hyperparameters. A popular swarm-intelligence-based algorithm is the particle swarm optimization (PSO) algorithm [15] which is well adapted to the optimization of nonlinear functions in multidimensional space. Differential evolution (DE) [16] has been particularly proposed for continuous search spaces and is very simple to implement. Vesterstrom and Thomsen [17] have evaluated the performance of GA, DE and PSO regarding their general applicability as numerical optimization techniques. Then, they concluded that DE is less sensitive to parameter changes than other metaheuristic algorithms. Therefore, the DE can rightfully be regarded as an excellent choice to hyperparameter optimization.

Memetic algorithm [18] is now widely used as a synergy of evolutionary or any population-based approach with separate individual learning or local improvement procedures for problem search. Evolution strategy (ES) is the first and oldest evolutionary algorithm, and it is based on the adaptation and evolution. Covariance matrix adaptation evolution strategies (CMA-ES) [19] is one of the most recent and powerful versions of memetic algorithm that combined evolution strategies with local information. The gene-pool optimal mixing evolutionary algorithm (GOMEA) is made for local search applying a strong mathematical background on the generation of the solutions, but it is considered to be a EA for discrete optimization problems [20]. Recently, Bouter et al. [21] proposed the real-valued GOMEA (RV-GOMEA) to cover the real-valued search space. Besides, memetic framework [22] models memetic algorithms as a process involving feature selection and learning procedure. In this paper, we present a wrapper-embedded memetic framework that utilizes DE to globally optimize the hyperparameters of non-convex regularization CHR that is a local search to select biomarkers in group.

**Fig 1:**
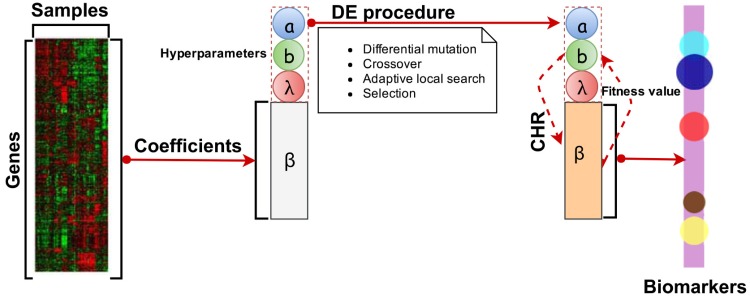
The workflow of our proposed the complex harmonic regularization with differential evolution algorithm (CHR-DE) for selecting biomarkers. Microarray gene expression data for one certain cancer are collected, processed into a matrix file that contains the genes (rows) and tissue samples (columns). In order to identify tumor subclasses that are both biologically meaningful and clinically relevant, we apply the differential evolution (DE) to fine tuning the hyperparameters of the complex harmonic regularization (CHR). After the operations of DE procedure, such as differential mutation, crossover, adaptive local search and selection, this CHR can be used in the learning procedures, and then give the fitness values feedback to update its hyperparameters. With a fully trained model, we can get some groups of genes with non-zero coefficients, which may be the valid biomarkers for this cancer.

The workflow of our proposed algorithm is shown in Fig 1. Microarray gene expression data for one certain cancer are collected, processed into a matrix file that contains the genes (rows) and tissue samples (columns). After setting the CHR’s hyperparameters in DE procedure, CHR starts the learning procedures, and then gives the fitness values feedback to update its hyperparameters. With a fully trained model, we can get some groups of genes with non-zero coefficients, which may be the valid biomarkers for this cancer.

The remainder of this paper is organized as follows: the CHR method for survival data in accelerated failure time (AFT) model is presented in Section 2, the implement of tuning CHR’s hyperparameters is introduced in Section 3, the experimental results and discussions are illustrated in Section 4, a concluding remark is finally made in Section 5.

## 2 Complex harmonic penalized accelerated failure time model

### 2.1 Accelerated failure time model

Suppose **X** denotes the *h* × *k* data matrix whose rows are *X*_*i*_ = (*x*_*i*1_, *x*_*i*2_, …, *x*_*ik*_), 1 ≤ *i* ≤ *h*, **T** denotes the sample vector of a lifetime or time to certain event of interest (*τ*_1_, *τ*_2_, …, *τ*_*h*_)^*T*^. Throughout this article we consider failure times (or survival times) that are right censored, survival time *τ*_*i*_ = *min*(*t*_*i*_, *c*_*i*_), where *t*_*i*_ is the true survival time, *c*_*i*_ is the time to the first censoring event (e.g., study conclusion, date of final follow up) for each subject *i*. Our survival data consist of independent observations for *h* individuals ${\left(\tau_{i},\;\delta_{i},\;X_{i}\right)}_{i=1}^{h}$, where *δ* is the censoring indicator, if *δ*_*i*_ = 0, it represents the right censoring time and *δ*_*i*_ = 1 means the completed time.

The accelerated failure time (AFT) model is treated as a linear regression between the survival time *τ*_*i*_ and the covariates *X*_*i*_: *G*(*τ*_*i*_) = *β*_0_ + *x*_*i*_ *β*^*T*^ + *ε*_*i*_, *i* = 1, 2, …, *h*, where G:[0,∞)→R, *β*_0_ is the intercept, $\beta \subseteq R^{k}$ is the regression coefficient, and *ε*_*i*_ are *h* independent random errors with a normal distribution function. Because of the censoring time in the datasets, the standard least squares approach is not allowed to directly compute the regression parameters of the covariates in AFT model.

In order to simplify the method, we use the mean imputation method [23] to estimate the right censored data in the least squares criterion. The estimated value *G*(*τ*_*i*_) of the censoring survival time *τ*_*i*_ is given by:

$$\begin{array}{c} G\left(\tau_{i}\right)=\delta_{i}\;log\left(\tau_{i}\right)+\left(1-\delta_{i}\right)\;{\left\{\hat{S}\left(\tau_{i}\right)\right\}}^{-1}\sum_{t_{\left(r\right)}>\tau_{i}}log\left(t_{\left(r\right)}\right)\Delta \hat{S}\left(t_{\left(r\right)}\right) \end{array}$$ (1)

where *t*_(⋅)_ are distinct censored lifetimes in an ascending sort order, *r* is the number of individuals at risk of failing just before time *t*(*i*), $\hat{S}$ is the Kaplan-Meier estimator [24] of the survival function, and $\Delta \hat{S}\left(t_{\left(r\right)}\right)$ is the step of $\hat{S}$ at time *t*_(*r*)_. Therefore, the least squares approach of AFT model is to minimize the loss function *L*(*β*) for the Gaussian family:

$$\begin{array}{c} L\left(\beta \right)=\frac{1}{h}\sum_{i=1}^{h}{\left(y_{i}-\sum_{j=0}^{k}\beta_{j}x_{ij}\right)}^{2} \end{array}$$ (2)

where the first column of **X** is all ones, and each censored *y*_*i*_ is replaced with the imputed value *G*(*τ*_*i*_).

### 2.2 Path seeking algorithm for complex harmonic regularization penalty

Regularization is a way to avoid over-fitting in AFT model and the common form of regularization for a control parameter λ (λ > 0) is:

$$\begin{array}{c} \hat{\beta }\left(\lambda \right)=\underset{\beta }{\arg\min}\{L(\beta )+\lambda P(\beta )\} \end{array}$$ (3)

where $\beta \in R^{p}$ are the estimated coefficients, *L*(*β*) is a loss function and *P*(*β*) represents the regularization term.

In fact, the survival data have different probability distributions of grouping effect and sparse. In theory, a strictly convex penalty function, such as *ℓ*_*q*_ (1 < *q* < 2), provides a sufficient condition for the grouping effect. On the contrary, *ℓ*_*p*_ (0 < *p* < 1) penalty can provide different sparse evaluation with different *p* value. The limitation of the existing regularization methods is that a fixed *p* (0 < *p* < 1) value *ℓ*_*p*_-norm with *ℓ*_2_-norm is used to evaluate the grouping effect and spares in variable selection, thus they often have assumptions about the probability distribution of the data. Upon our previous work naïve harmonic regularization that can approximate $\ell_{p}\;\left(\frac{1}{2}\leq p<1\right)$ penalties [6], we designed the CHR penalty that can approximate the combination of the $\ell_{p}\;\left(\frac{1}{2}\leq p<1\right)$ and *ℓ*_*q*_ (1 ≤ *q* < 2) penalties [10]. The CHR penalty can be normally expressed as:

$$\begin{array}{c} \hat{\beta }=\arg\min_{\beta }\{L\left(\beta \right)+\lambda_{1}\sum_{j=1}^{k}m\left(\beta_{j}\right)+\lambda_{2}\sum_{j=1}^{k}n\left(\beta_{j}\right)\} \end{array}$$ (4)

where 0 < *a*, *b* < 1; λ_1_, λ_2_ ≥ 0;

$$\begin{array}{cc} m(\cdot ) & =\sqrt{\frac{2}{a(a+1)}|\cdot |+{\left(\frac{1-a}{a}\right)}^{2}}-\frac{1-a}{a};\\ n(\cdot ) & =\sqrt{\frac{2}{b(b+1)}{\left|\cdot \right|}^{2}+{\left(\frac{1-b}{b}\right)}^{2}}-\frac{1-b}{b}. \end{array}$$

**Fig 2:**
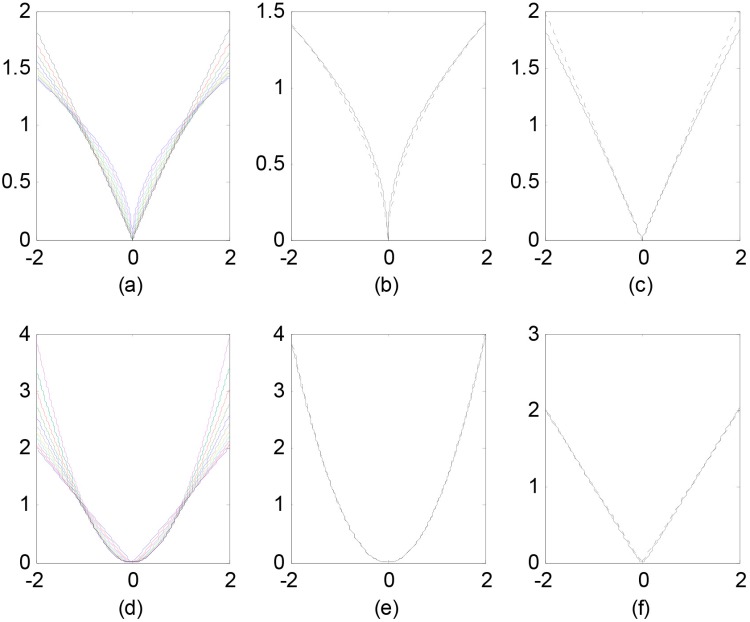
The complex harmonic regularization. (a) the curves represent *m*(⋅) at different parameter *a* values; (b) the solid curve represents *m*(⋅) at the parameter *a* = 0.99, and the dashed curve is the *ℓ*_1/2_ regularization; (c) the solid curve represents *m*(⋅) at the parameter *a* = 0.01, and the dashed curve is the *ℓ*_1_ regularization; (d) the curves represent *n*(⋅) at different parameter *b* values; (e) the solid curve represents *n*(⋅) at the parameter *b* = 0.01, and the dashed curve is the *ℓ*_2_ regularization; (f) the solid curve represents *n*(⋅) at the parameter *b* = 0.99, and the dashed curve is the *ℓ*_1_ regularization.

Furthermore, comparing with the fixed *p* and *q*, the CHR penalty can suggest a proper value for *p* and *q* in given datasets, and the CHR penalty can be plotted as Fig 2. When *a* is close to 0, *m*(*β*) ≈ |*β*| (*ℓ*_1_-norm, see Fig 2(c)). When *a* is close to 1, $m\left(\beta \right)=\sqrt{\left|\beta \right|}$ (*ℓ*_1/2_-norm, see Fig 2(b)). When *b* is close to 0, *n*(*β*) ≈ |*β*|^2^ (*ℓ*_2_-norm, see Fig 2(e)). When *b* is close to 1, *n*(*β*) = |*β*| (see Fig 2(f)), that is same with *a* closing to 0.

**Theorem 1**. *m*(⋅) *and n*(⋅) *approximate to the combination of* $\ell_{p}\;\left(\frac{1}{2}\leq p<1\right)$ *and ℓ*_*q*_ (1 ≤ *q* < 2) *regularizations with adjustable p and q to evaluate the grouping effect and sparse of data, i.e*.,

$$\begin{array}{c} \lim_{a\to 0}m\left(\beta \right)\approx |\beta |\;(Lasso),\;\lim_{a\to 1}m\left(\beta \right)=\sqrt{\left|\beta \right|}\;\left(\ell_{1/2}\right),\\ \lim_{b\to 0}n\left(\beta \right)\approx {\left|\beta \right|}^{2}\;\left(\ell_{2}\right),\;\lim_{b\to 1}n\left(\beta \right)=|\beta |. \end{array}$$

*Proof*.

$$\begin{array}{cc} \lim_{a\to 0}m\left(\beta \right) & =\lim_{a\to 0}\sqrt{\frac{2}{a(a+1)}|\beta |+{\left(\frac{1-a}{a}\right)}^{2}}-\frac{1-a}{a}\\ & =\lim_{a\to 0}\frac{\sqrt{\frac{2a}{\left(a+1\right){\left(1-a\right)}^{2}}|\beta |+1}-1}{\frac{a}{1-a}}\\ & =\lim_{a\to 0}\frac{1+\frac{2a}{2\left(a+1\right){\left(1-a\right)}^{2}}|\beta |+o\left({\left(\frac{2a}{\left(a+1\right){\left(1-a\right)}^{2}}|\beta |\right)}^{2}\right)-1}{\frac{a}{1-a}}\\ & =\lim_{a\to 0}\frac{1}{\left(a+1)(1-a\right)}|\beta |+o\left({\left(\frac{2}{\left(a+1)(1-a\right)}|\beta |\right)}^{2}\right)\\ & \approx |\beta |\\ \lim_{a\to 1}m\left(\beta \right) & =\lim_{a\to 1}\sqrt{\frac{2}{a(a+1)}|\beta |+{\left(\frac{1-a}{a}\right)}^{2}}-\frac{1-a}{a}\\ & =\sqrt{\left|\beta \right|} \end{array}$$

There are the inductions of the first two equations. The inductions of other two equations are similar to these and need not be explained here.

Let $\gamma =\frac{\lambda_{1}}{\lambda_{1}+\lambda_{2}},\;\lambda =\lambda_{1}+\lambda_{2}$ in Eq (4), then the common form of CHR penalty can be re-expressed as:

$$\begin{array}{c} \hat{\beta }=\arg\min_{\beta }\{L\left(\beta \right)+\lambda (\gamma \sum_{j=1}^{k}m\left(\beta_{j}\right)+\left(1-\gamma \right)\sum_{j=1}^{k}n\left(\beta_{j}\right))\} \end{array}$$ (5)

Therefore, we can use the path seeking algorithm [25] in linear model to sequentially construct a path directly in parameter space that closely approximates that for CHR penalty, without having repeatedly solve numerical optimization problem.

Let *ν* measure length along the path and Δ*ν* > 0 be a *small* increment. Here, we need to note that the size of the step Δ*ν* can be obtained by

$$\begin{array}{c} \frac{L\left(\hat{\beta }\left(\nu \right)\right)-L\left(\hat{\beta }\left(\nu +\Delta \nu \right)\right)}{L(\hat{\beta }\left(\nu \right))}=0.01 \end{array}$$ (6)

Define

$$\begin{array}{cc} \varphi_{j}\left(\nu \right) & =-{\left[\frac{\partial L(\beta )}{\partial \beta_{j}}\right]}_{\beta =\hat{\beta }\left(\nu \right)}\\ & =-{\left[\frac{\partial \frac{1}{h}\sum_{i=1}^{h}{\left(y_{i}-\sum_{j=0}^{k}\beta_{j}x_{ij}\right)}^{2}}{\partial \beta_{j}}\right]}_{\beta =\hat{\beta }\left(\nu \right)}\\ & ={\left[\frac{2}{h}\sum_{i=1}^{h}x_{ij}\left(y_{i}-\sum_{j=0}^{k}\beta_{j}x_{ij}\right)\right]}_{\beta =\hat{\beta }\left(\nu \right)} \end{array}$$ (7)

$$\begin{array}{cc} \phi_{j}\left(\nu \right) & ={\left[\frac{\partial (\gamma \sum_{j=1}^{k}m\left(\beta_{j}\right)+\left(1-\gamma \right)\sum_{j=1}^{k}n\left(\beta_{j}\right))}{\partial |\beta_{j}|}\right]}_{\beta =\hat{\beta }\left(\nu \right)}\\ & ={\left[\frac{\gamma }{\sqrt{2a\left(a+1\right)|\beta_{j}|+{\left(1-a^{2}\right)}^{2}}}+\frac{2\left(1-\gamma \right)|\beta_{j}|}{\sqrt{2b\left(b+1\right){\left|\beta_{j}\right|}^{2}+{\left(1-b^{2}\right)}^{2}}}\right]}_{\beta =\hat{\beta }\left(\nu \right)} \end{array}$$ (8)

$$\begin{array}{cc} \lambda_{j}\left(\nu \right) & =\frac{\varphi_{j}\left(\nu \right)}{\phi_{j}\left(\nu \right)} \end{array}$$ (9)

where λ_*j*_(*ν*) is the ratio of these two gradients *φ*_*j*_(*ν*) for loss function Eq (2) and *ϕ*_*j*_(*ν*) for the penalty function with respect to |*β*_*j*_|. This path seeking scheme can accelerate solving the CHR penalty. The details of the implementation of CHR penalty are outlined in Algorithm 1.

**Algorithm 1** Implementation of CHR penalty

1: Initialize: $\nu =0,{\left\{{\hat{\beta }}_{j}\left(0\right)=0\right\}}_{1}^{k}$

2: **repeat**

3:  Compute ${\left\{\lambda_{j}\left(\nu \right)\right\}}_{1}^{k}$

4:  $S=\{j|\lambda_{j}\left(\nu \right)\cdot \hat{\beta }\left(\nu \right)<0\}$

5:  **if** *S* = empty **then**

6:   j* = arg max_*j*_ |λ_*j*_(*ν*)|

7:  **else**

8:   j* = arg max_*j*∈*S*_ |λ_*j*_(*ν*)|

9:  **end if**

10:  ${\hat{\beta }}_{j^{*}}\left(\nu +\Delta \nu \right)={\hat{\beta }}_{j^{*}}\left(\nu \right)+\Delta \nu \cdot sign\left(\lambda_{j^{*}}\left(\nu \right)\right)$

11:  ${\left\{{\hat{\beta }}_{j}\left(\nu +\Delta \nu \right)={\hat{\beta }}_{j}\left(\nu \right)\right\}}_{j\neq j^{*}}$

12:  *ν* ← *ν* + Δ*ν*

13: **untill** λ(*ν*) = 0

After initializing the path, the vector λ(*ν*) is computed via Eqs (7)–(9) at each step. Then, those non zero coefficients $\hat{\beta }\left(\nu \right)\neq 0$ which have a sign opposite to that of their corresponding λ_*j*_(*ν*) are identified. When the set *S* is empty, the coefficient corresponding to the largest component of λ(*ν*), in absolute value is selected at line 6. And when there are one or more elements in the set *S*, the coefficient with corresponding largest |λ_*j*_(*ν*)| within this subset is instead selected. The selected coefficient ${\hat{\beta }}_{j^{*}}\left(\nu \right)$ is then incriminated by a small amount in the direction of the sign of its correspond λ_j*_(*ν*) with all other coefficient remaining unchanged, producing the solution for the next path point *ν* + Δ*ν*. Iterations continue until all components of λ(*ν*) are zero.

Although the complex harmonic penalized AFT model can adapt for different data distributions, this model has three hyperparameters *a*, *b*, *γ* which are sensitive to the resolution. The more suitable way thereby is optimized by the evolutionary algorithms to make these regularized hyperparameters more precise and efficient.

## 3 Complex harmonic regularization in a memetic framework

### 3.1 A wrapper-embedded memetic framework

Memetic framework [22] models memetic algorithms (MAs) as a process involving feature selection and learning procedure. The term of MAs, which combine evolutionary algorithms (EAs) with local search (LS) [26], have recently received much attention from the feature selection problems. These methods are inspired by Darwin’s principles of natural evolution and Dawkins defined memes, which unlike genes, can adapt themselves [27].

In most memetic-based feature selection approaches, an EA is used for wapper feature selection and a LS algorithm is used for filter feature selection. Zhu et al [28] applied genetic algorithm for wrapper feature selection and used Markov blanket approach as a LS for filter feature selection. Noman and Iba [29] incorporated a crossover-based LS with adaptive length in DE resulted into a DE-variant, where the length of the LS algorithm can be adjusted adaptively using a hill climbing heuristic. However, such memetic-based approaches have the potential limitation that filter evaluation measures may eliminate potentially useful features regardless of their performance in the wrapper approaches. In addition, the wrapper approaches usually involve a large number of assessments, and each assessment usually takes a considerable amount of time, especially when the numbers of features and instances are large. The second limitation of the existing memetic-based feature selection methods is that they are primarily concerned with the relatively small numbers of features and instances.

Focusing on these limitations above, regularization method can adapting relationships between data by designing different penalty functions with original, grouping effect or net effect. What’s more, regularization methods evaluate features and build model at one stage. Therefore, we embed CHR penalty into a DE-variant for improving the selection ability under the global optimization of the non-convex regularization.

### 3.2 Implementation of complex harmonic regularization with differential evolution (CHR-DE) algorithm

Our proposed wrapper-embedded feature selection approach (CHR-DE) in memetic framework includes population-initialized, differential mutation, crossover, adaptive local search and selection operations. The first step of the CHR-DE approach is that the DE population is randomly initialized with each chromosome encoding the penalized hyperparameters (intron) and the coefficients of each gene in the AFT model (exon). Subsequently, the CHR approach (local search) is performed on the exon part under the fixed intron part, to reach a local optimal solution or to improve the fitness of individuals in the search population. DE operations are performed on the intron parts of the chromosomes, and the selection operator generates the next population. This process repeats itself till the stopping conditions are satisfied. The details of this approach are outlined in Algorithm 2.

**Algorithm 2** The CHR-DE algorithm in memetic framework

**Input**:

 Bounds of solution space *h*_*b*_, *l*_*b*_;

 Population size *N*_*P*_;

 Individual size *N*_*D*_;

 Fitness function *f*(⋅); //Embedded with CHR penalty

 Crossover rate *cr*;

 Scaling factor *F*;

**Output**: Regression coefficient β*.

1: Generate initial population //Begin DE procedure

2: *pop* ← *rand*(*N*_*P*_, *N*_*D*_) × (*h*_*b*_ − *l*_*b*_) + *l*_*b*_

3: **for** *i* = 1: *N*_*P*_ **do**

4:  Calculate *f*(*pop*(*i*))

5: **end for**

6: **repeat**

7:  Select *pop*_*r*_, *pop*_*s*_ *pop*_*t*_ randomly in *pop*

8:  //Differential mutation

9:  **for** *i* = 1: *N*_*P*_ **do**

10:   *child*(*i*) ← *pop_r_* + *F* × (*pop_s_* + *pop_t_*)

11:   //Crossover

12:   *j*_*rand*_ = ⌊*rand* × *N*_*D*_⌋

13:   **for** *j* = 1: *N*_*D*_ **do**

14:    **if** *rand* < *cr* OR *j* == *j*_*rand*_ **then**

15:     *offspring*(*i*)(*j*) ← *child*(*i*)(*j*)

16:    **else**

17:     *offspring*(*i*)(*j*) ← *pop*(*i*)(*j*)

18:    **end if**

19:   **end for**

20:   //Selection

21:   **if** *f*(*offspring*) ≥ *f*(*pop*) **then**

22:    *pop* ← *offspring*

23:   **end if**

24:  **end for**

25:  //Adaptive local search

26:  *tmpPop* ← *mean*(*pop*) + *w*_*L*_(*pop* − *mean*(*pop*))

27:  **for** *i* = 1: *N*_*P*_ **do**

28:   **for** *j* = 1: *N*_*P*_ − 1 **do**

29:    $r\left(j\right){rand}^{\frac{1}{j+1}}$

30:   **end for**

31:   *C*(1) ← 0

32:   **for** *j* = 2: *N*_*P*_ **do**

33:    *C*(*j*) ← *r*(*j* − 1)(*tmpPop*(*i* − 1) − *tmpPop*(*i*) + *C*(*j* − 1))

34:   **end for**

35:   *offspring* ← *tmpPop*(*N_P_*) + *C*(*N_P_*)

36:   **if** *offspring* ∈ (*h_b_*, *l_b_*) AND *f*(*offspring*) ≥ *f*(*pop*(*i*)) **then**

37:    *pop*(*i*) ← *offspring*

38:   **end if**

39:  **end for**

40: **untill** stopping criterion is met

#### 3.2.1 Chromosome representation: Intron and exon

The first step of the CHR-DE approach is that the population of *N*_*P*_ individuals initializing randomly with each chromosome which adopts the “intron + exon” encoding [13] to construct the penalized hyperparameters (intron) and the coefficients of each gene in the AFT model (exon), i.e., *c* = (*a*, *b*, *γ*, *β*_1_, *β*_2_, ⋯, *β*_*k*_). In CHR scheme, there are three parameters in intron part $pop={\left[a,b,\gamma \right]}_{1}^{N_{P}}$ which should cover this range by uniformly randomizing individuals with minimum and maximum bounds *l*_*b*_, *h*_*b*_ in the search space. DE searches for a global optimum in intron part which is *N*_*D*_ dimensional real parameter space $R^{N_{D}}$:

$$\begin{array}{c} pop=rand\left(N_{P},\;N_{D}\right)\times \left(h_{b}-l_{b}\right)+l_{b} \end{array}$$ (10)

where *rand* is a uniformly distributed random number lying between 0 and 1. Meanwhile, the CHR is performed on exon part for each introns in individuals, i.e., *β* to reach a local optimal solution and to gain the fitness of each individuals.

#### 3.2.2 Fitness definition

The mean squared error (MSE) and the concordance index (CI) are two criteria used to design a fitness function. In statistics, the MSE measures the average of the squares of the errors, which is evaluated by Eq (11) for survival data.

$$\begin{array}{c} mse\left(\beta \right)=\frac{1}{h}\sum_{i=1}^{h}{\left(\tau_{i}-{\hat{\tau }}_{i}\right)}^{2} \end{array}$$ (11)

where the predicted value ${\hat{\tau }}_{i}=exp\left(\sum_{j=0}^{k}\beta_{j}x_{ij}\right)$.

In survival analysis, the CI is the standard performance measure for model assessment and quantifies the quality of rankings by Eq (12).

$$\begin{array}{c} ci\left(\beta \right)=\frac{\sum_{i}\sum_{j}\;1\;\left({\hat{\tau }}_{i}<{\hat{\tau }}_{j}\;\text{and}\;\delta_{i}=1\right)}{\sum_{i}\sum_{j}\;1\;\left(\tau_{i}<\tau_{j}\;\text{and}\;\delta_{i}=1\right)} \end{array}$$ (12)

We employ the weighted-sum method [30] to change this bi-objective problem into a single objective problem. Thus, the individual with low MSE and high CI produces a high fitness value by Eq (13).

$$\begin{array}{c} fitness_{i}=w_{M}\times \left(1-\frac{MSE_{i}}{\sum_{i=1}^{N_{P}}MSE_{i}}\right)+w_{C}\times CI_{i} \end{array}$$ (13)

where *w*_*M*_ is the weight of MSE for the individual *i* in the population, *w*_*C*_ is the CI for this individual. These weight factors can be adjusted according to what people value as an important weight, e.g., if MSE is more important than CI, we set the weight factors *w*_*M*_ = 95%, *w*_*C*_ = 5%. Furthermore, the results with different values of *w*_*M*_ and *w*_*C*_ can be found in the S1 Appendix.

#### 3.2.3 Differential mutation operation

After initialization, DE uses a differential mutation operator based on linear combination.

$$\begin{array}{c} child=pop_{r}+F\times \left(pop_{s}+pop_{t}\right) \end{array}$$ (14)

The indices *r*, *s*, *t* are mutually exclusive integers randomly generated within the range [1, *N*_*P*_]. These indices are randomly generated once for each mutant vector *child*. The scaling factor *F* ∈ [0, 1+[ is a positive value which cannot be much greater than 1 for scaling the difference vector [31].

#### 3.2.4 Crossover operation

To enhance the potential diversity of the population, a crossover operation applied to each pair of the target vector *pop* and its corresponding mutant vector *child* to generate a trial vector *offspring*. We employ the binomial (uniform) crossover to create a single trial vector. This crossover is defined for each *j*th component of the *i*th parameter vector as follows:

$$\begin{array}{c} {\text{offspring}}_{i,j}=\{\begin{array}{cc} & child_{i,j}\;\;\;\text{if}\;rand<cr\;\text{or}\;j=j_{rand}\\ & pop_{i,j}\;\;\;\;\;\text{otherwise} \end{array} \end{array}$$ (15)

where *j*_*rand*_ ∈ [1, 2, ⋯, *N*_*D*_] is a randomly chosen index, which ensures that *offspring* gets at least one component from *child*.

#### 3.2.5 Adaptive local search

Usually in EAs the solutions with better fitness values are generally for reproduction, thus we use adaptive simplex crossover local search strategy for exploring the neighborhood of the best individual of population. Firstly, we expand the population with simplex crossover:

$$\begin{array}{c} tmpPop=mean\left(pop\right)+w_{L}\left(pop-mean\left(pop\right)\right) \end{array}$$ (16)

where *w*_*L*_ is the control parameter of this local search. Then, generating the offspring upon the expansion population in Eqs (17) and (18).

$$Ci=\{\begin{array}{c} \begin{array}{cc} 0, & \left(i=1\right) \end{array}\\ r_{i-1}\left(tmpPop_{i-1}-tmpPop_{i}+C_{i-1}\right),\left(i=2,\cdots ,N_{p}\right) \end{array}$$ (17)

$$\begin{array}{c} \text{offspring}=tmpPop_{N_{P}}+C_{N_{P}} \end{array}$$ (18)

#### 3.2.6 Selection operation

The solutions with better fitness values are generally preferred for reproduction, as they are more likely to be in the proximity of a basin of attraction. Therefore, we deterministically select the best individual of the population for exploring its neighborhood using the selection operation that is described as

$$\begin{array}{c} pop=\{\begin{array}{cc} \text{offspring} & \text{if}\;f(\text{offspring})\geq f(pop)\\ pop & \text{otherwise} \end{array} \end{array}$$ (19)

where *f*(⋅) is the fitness function in Eq (13) to be maximized. Therefore, if the new trial vector yields an equal or higher value of the fitness function, it replaces the corresponding target vector in the next generation; otherwise the target is retained in the population. Hence, the population either gets better or remains the same in fitness status, but never deteriorates.

## 4 Results and discussion

### 4.1 Synthetic datasets

To demonstrate the performance of our proposed regularization procedure, we assume that the graph modules with 200 key factors (KFs) and that each regulates 10 different genes for a total of 2200 variables. Among these models and genes, 4 KFs and their 10 regulated genes (44 variables in total) are associated with the response based on the following model:

$$\begin{array}{c} Y=\sum_{u=1}^{44}\beta_{u}X_{u}+\varepsilon \end{array}$$ (20)

where the independent random noise *ε* ∼ *N*(0, 1), and the non-zero coefficients are specified as

$$\begin{array}{c} \beta_{u}=\left(2,\underset{10}{\underset{︸}{\frac{2}{\sqrt{10}},\cdots ,\frac{2}{\sqrt{10}}}},-2,\underset{10}{\underset{︸}{\frac{-2}{\sqrt{10}},\cdots ,\frac{-2}{\sqrt{10}}}},4,\underset{10}{\underset{︸}{\frac{4}{\sqrt{10}},\cdots ,\frac{4}{\sqrt{10}}}},-4,\underset{10}{\underset{︸}{\frac{-4}{\sqrt{10}},\cdots ,\frac{-4}{\sqrt{10}}}}\right). \end{array}$$

For each KF, the **X** value is simulated from a *N*(0, 1) distribution, and conditional on the value of KF, we simulate the expression levels of the genes that they regulated from a conditional normal distributions *ϱ* of 0.2, 0.5, 0.7, and 0.9, respectively. For example, if the *x*_1_ is KF of *x*_*i*_, *i* = 2, 3, ⋯, 10, then we can define this group is *x_i_* = *ϱ* × *x*_1_ + (1 − *ϱ*) × *x_i_*. Therefore, we have a total of 2200 variables and 44 of them are relevant.

All of penalties in our experiments are solved by the general path seeking method [25]. The original DE for feature subset selection was conducted by Khushaba et al. [32]. For each model, we use two-thirds of simulated data for training and remaining one-third for testing with 600 samples. A 10-fold cross validation (CV) is conducted on training set for tuning parameters of all approaches. In our experimentation, the scaling factor *F* = 0.9, cross rate *cr* = 0.9, and the weight factors *w*_*M*_ = 95%, *w*_*C*_ = 5%, *w*_*L*_ = 1 respectively. Because the population size should be small [29], we set *N*_*P*_ = 4, and the stoping criterion of 10,000. In addition, we also calculate both *sensitivity* and *specificity* for each procedure, where

$$\begin{array}{cc} sensitivity & =\frac{\#\;\text{correctly}\;\text{selected}\;\text{genes}}{\#\;\text{non-zero}\;\text{in}\;\beta_{u}}\\ & =\frac{\#\;\text{correctly}\;\text{selected}\;\text{genes}}{44} \end{array}$$ (21)

$$\begin{array}{cc} specificity & =\frac{\#\;\text{correctly}\;\text{rejected}\;\text{genes}}{\#\;\text{zero}\;\text{in}\;\beta_{u}}\\ & =\frac{\#\;\text{correctly}\;\text{rejected}\;\text{genes}}{2200-44} \end{array}$$ (22)

To further evaluate the performance of each penalties, we employ the prediction mean-squared errors (MSE) and the concordance index (CI) with standard errors.

After repeating the each penalties 50 times, the averaged results are summarized in Table 1. Generally, our proposed CHR-DE approach gives lower MSE with higher CI than other approaches. The CHR-DE also results in much higher sensitivity with comparable specificity for identifying the relevant features. The Lasso and *ℓ*_1/2_ without *ℓ*_2_-norm have strong selectivity especially in high grouping effect data *ϱ* = 0.7, 0.9. With the correlation *ϱ* increasing among genes, these no grouping effect penalties select a few genes, e.g., the sensitivity of *ℓ*_1/2_ is from 0.790 down to 0.091 (only selecting these 4 non-zero coefficient KFs) with highest specificity 0.998. The wrapper methods DE and CMA-ES have weaker selectivity than other grouping effect penalties, e.g., Elastic net, *ℓ*_1/2_ + *ℓ*_2_ and CHR, especially in the data containing low correlation features *ϱ* = 0.2. Although other grouping effect penalties have lower specificity, they perform well and select more correct genes whose coefficients *β* is non-zero, no matter what the conditional normal distributions *ϱ*. Comparing with the CHR’s hyperparameters tuning by grid search (CHR-GS), the CHR-DE utilizes the evolutionary algorithm to skip redundant parameter settings or to add new ones and ultimately achieves better performance.

**Table 1:** Results of the synthetic data, sensitivity, specificity, mean-squared-error (MSE), concordance index (CI) are based on 50 simulations. Standard errors are given in parentheses.

*ϱ*	Penalty	Sensitivity	Specificity	MSE	CI
0.2	Lasso	0.863 (0.152)	0.996 (0.013)	21.911 (3.268)	0.841 (0.018)
	*ℓ*_1/2_	0.790 (0.082)	**0.998** (0.001)	11.318 (2.131)	0.849 (0.016)
	DE	0.809 (0.066)	0.990 (0.012)	20.451 (1.875)	0.879 (0.017)
	CMA-ES	0.746 (0.063)	0.986 (0.015)	20.786 (2.666)	0.861 (0.016)
	Elastic net	0.840 (0.164)	0.936 (0.014)	8.649 (1.918)	0.883 (0.026)
	*ℓ*_1/2_ + *ℓ*_2_	0.922 (0.136)	0.953 (0.016)	6.777 (1.754)	0.901 (0.016)
	CHR-GS	0.977 (0.066)	0.956 (0.015)	6.746 (1.713)	0.912 (0.014)
	CHR-DE	**0.988** (0.081)	0.962 (0.012)	**6.461** (1.520)	**0.914** (0.012)
0.5	Lasso	0.795 (0.117)	0.996 (0.013)	21.615 (3.380)	0.880 (0.023)
	*ℓ*_1/2_	0.272 (0.052)	**0.998** (0.001)	11.475 (2.462)	0.929 (0.034)
	DE	0.871 (0.071)	0.992 (0.013)	18.518 (2.924)	0.946 (0.029)
	CMA-ES	0.735 (0.067)	0.986 (0.016)	18.614 (2.132)	0.949 (0.025)
	Elastic net	0.818 (0.183)	0.928 (0.015)	9.605 (2.764)	0.961 (0.035)
	*ℓ*_1/2_ + *ℓ*_2_	0.886 (0.167)	0.931 (0.014)	9.391 (3.479)	0.966 (0.028)
	CHR-GS	0.928 (0.035)	0.947 (0.017)	9.375 (2.466)	0.969 (0.023)
	CHR-DE	**0.931** (0.054)	0.949 (0.013)	**8.031** (2.357)	**0.972** (0.027)
0.7	Lasso	0.681 (0.023)	0.997 (0.014)	29.822 (2.945)	0.882 (0.022)
	*ℓ*_1/2_	0.091 (0.003)	**0.998** (0.001)	22.850 (2.397)	0.945 (0.028)
	DE	0.720 (0.039)	0.991 (0.012)	15.727 (2.628)	0.963 (0.030)
	CMA-ES	0.680 (0.028)	0.987 (0.016)	16.017 (1.922)	0.966 (0.025)
	Elastic net	0.863 (0.192)	0.853 (0.015)	12.873 (2.521)	0.977 (0.031)
	*ℓ*_1/2_ + *ℓ*_2_	0.841 (0.133)	0.882 (0.010)	13.351 (2.757)	0.965 (0.028)
	CHR-GS	0.923 (0.032)	0.903 (0.011)	12.560 (1.711)	**0.978** (0.024)
	CHR-DE	**0.946** (0.061)	0.924 (0.008)	**12.452** (1.188)	**0.978** (0.023)
0.9	Lasso	0.409 (0.005)	0.995 (0.013)	34.439 (2.113)	0.878 (0.027)
	*ℓ*_1/2_	0.091 (0.003)	**0.998** (0.001)	29.565 (1.798)	0.935 (0.025)
	DE	0.697 (0.046)	0.990 (0.012)	15.151 (2.757)	0.967 (0.028)
	CMA-ES	0.435 (0.011)	0.986 (0.018)	15.513 (2.077)	0.965 (0.025)
	Elastic net	0.727 (0.153)	0.824 (0.016)	23.764 (2.463)	0.941 (0.034)
	*ℓ*_1/2_ + *ℓ*_2_	0.795 (0.126)	0.831 (0.012)	15.478 (2.826)	0.967 (0.030)
	CHR-GS	0.864 (0.082)	0.844 (0.009)	14.113 (1.523)	0.976 (0.026)
	CHR-DE	**0.909** (0.063)	0.873 (0.006)	**13.351** (1.182)	**0.977** (0.024)

### 4.2 Real datasets

**Fig 3:**
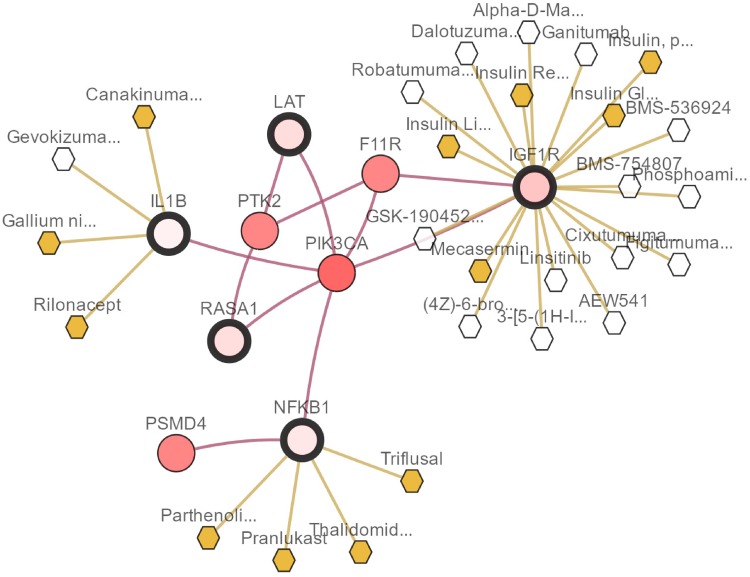
The network views of IL1B, NFKB1, IGF1R, LAT and RASA1 in the breast cancer rendered with cBioPortal [35]. The selected genes by CHR-DE are outlined with a thick border, and all other genes are automatically identified as altered in one cancer. Darker red indicates increased frequency of alteration (defined by mutation, copy number amplification, or homozygous deletion) in one cancer. The drugs that target genes are display with hexagons, and orange indicates FDA-approved.

**Fig 4:**
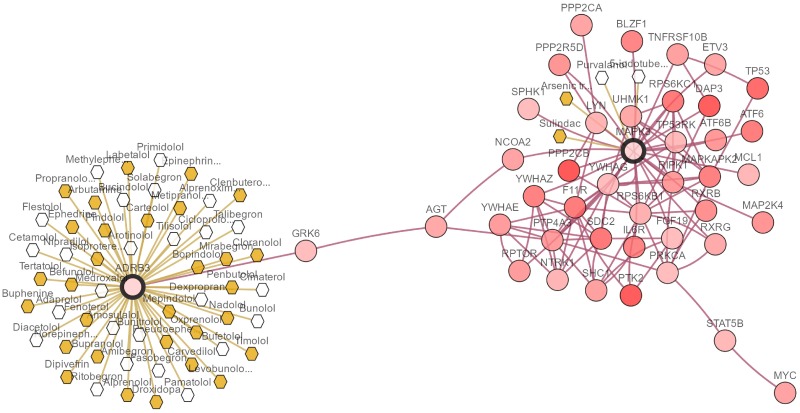
The network view of ADRB3 and MAPK3 in the hepatocellular carcinoma rendered with cBioPortal [35]. The selected genes by CHR-DE are outlined with a thick border, and all other genes are automatically identified as altered in one cancer. Darker red indicates increased frequency of alteration (defined by mutation, copy number amplification, or homozygous deletion) in one cancer. The drugs that target genes are display with hexagons, and orange indicates FDA-approved.

**Fig 5:**
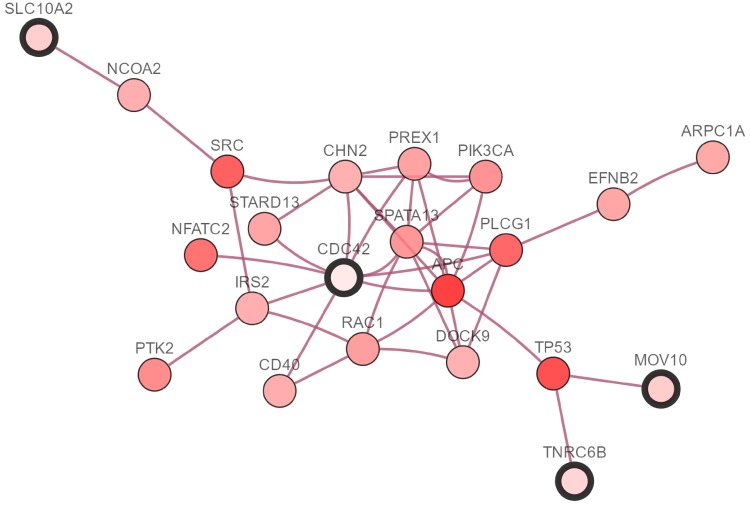
The network view of CDC42, SLC10A2, TNRC6B and MOV10 in the colorectal cancer rendered with cBioPortal [35]. The selected genes by CHR-DE are outlined with a thick border, and all other genes are automatically identified as altered in colorectal cancer. Darker red indicates increased frequency of alteration (defined by mutation, copy number amplification, or homozygous deletion) in one cancer.

We demonstrate the proposed methods by analyzing microarray expression data from NCBI’s gene expression omnibus (GEO) with the accession number, including breast cancer (GSE22210) [33], hepatocellular carcinoma (HCC, GSE10141) [34] and colorectal cancer (CRC, GSE103479). To evaluate our CHR-DE method, we divide these datasets at random two-thirds samples become training set and the remainders are test set. The details about these above datasets are shown in Table 2. Besides, the Figs 3–5 show the pathways of some selected genes by CHR-DE method in three different cancers rendered with cBioPortal [35]. The query genes are outlined with a thick border, and all other genes are automatically identified as altered in one cancer. Darker red indicates increased frequency of alteration (defined by mutation, copy number amplification, or homozygous deletion) in one cancer. The drugs that target genes are display with hexagons, and orange indicates FDA-approved.

**Table 2:** The real datasets.

Dataset	# genes	# samples (training / test)
GSE22210	1,452	167 (117 / 50)
GSE10141	6,144	80 (56 / 24)
GSE103479	110,961	155 (109 / 46)

#### 4.2.1 Breast cancer

GSE22210 contains 167 breast tumor samples with 1,452 genes obtained using GEO Platform GPL9183 [33]. Table 3 shows that the CHR-DE performs best in predicting the patients’ survival time with selecting smaller number of genes than the Elastic net and CHR-GS.

**Table 3:** The results with standard errors in parentheses for GSE22210.

Penalty	# selected genes	MSE	CI
Lasso	46	21.023 (2.680)	0.776 (0.017)
*ℓ*_1/2_	23	25.271 (2.432)	0.783 (0.019)
DE	44	21.421 (2.381)	0.735 (0.024)
CMA-ES	38.5	28.835 (2.619)	0.695 (0.016)
Elastic net	159	33.331 (2.125)	0.809 (0.025)
*ℓ*_1/2_ + *ℓ*_2_	104.333	31.975 (1.992)	0.805 (0.022)
CHR-GS	122.667	18.790 (1.987)	0.813 (0.014)
CHR-DE	117.667	**17.371** (1.871)	**0.815** (0.015)

As see from the Table 4, CHR-DE penalty selects some unique genes, such as **HIC1** **LIF** which play an important role in the development of primary breast cancer [36, 37]. The **XIST** is selected by these 8 different methods and lack an X chromosome decorated by XIST RNA causes the basal-like subtype of invasive breast carcinoma [38]. Moreover, some relevant genes are selected by other regularization models such as IL1B, NFKB1, IGF1R and SERPINB2 which are also found by the CHR-DE. Especially, the IL1B, NFKB1 and IGF1R in a small group of network by CHR-DE method as shown in Fig 3, and they are also targeted by several cancer drugs. The **IL1B** leads to enhanced production of proinflammatory cytokines triggered by the treatment, with subsequent effects on persistent fatigue in the aftermath of breast cancer [39]. Wood et al [40] identified **NFKB1** mutation in breast tumorigenesis. As one of related receptors in insulin-like growth factor (IGF) system, type I IGF receptor (**IGF1R**) can influence the activity of estrogen receptor-*α* (ER) that can be used in promoting breast tumor regression [41]. The the plasminogen activator inhibitor type 2 (PAI2, **SERPINB2**), is significantly associated with increased survival in patients with breast cancer [42, 43].

**Table 4:** The top 10 selected genes in the GSE22210.

	Lasso	*ℓ*_1/2_	DE	CMA-ES	Elastic net	*ℓ*_1/2_ + *ℓ*_2_	CHR-GS	CHR-DE
1	XIST	IL1B	IGSF4C	ASB4	SERPINB2	XIST	XIST	SERPINB2
2	LAT	XIST	AFF3	KIAA1804	XIST	IL1B	IL1B	IMPACT
3	IL1B	HLA-DQA2	BMP4	CASP10	IMPACT	LAT	LAT	XIST
4	DNASE1L1	TGFA	IGF2AS	CDKN2A	IL1B	ESR2	NFKB1	HIC1
5	NFKB1	CDKN1A	XIST	TERT	LAT	KCNK4	TGFA	IGF1R
6	HDAC9	GNMT	CD9	BCAP31	CCND1	IGF1R	CDKN1A	LAT
7	BCL2L2	LAT	CDC25B	GLI2	NFKB1	CD1A	RASA1	LIF
8	ESR2	BCL2L2	MMP1	XIST	TGFA	PTPRF	HDAC9	IL1B
9	AFP	HDAC9	NFKB2	ABCG2	HLA-DQA2	HLA-DQA2	LAMC1	NFKB1
10	LAMC1	CD44	HFE	CCKBR	RASGRF1	TGFA	RASGRF1	RASA1

#### 4.2.2 Hepatocellular carcinoma

GSE10141 contains 6,144 genes for 80 hepatocellular carcinoma (HCC) patients. Table 5 also shows that the CHR-DE performed best in predicting the patients’ survival time with selecting smaller number of genes than the Elastic net and CHR-GS.

**Table 5:** The results with standard errors in parentheses for GSE10141.

Penalty	# selected genes	MSE	CI
Lasso	29	31.228 (3.165)	0.764 (0.030)
*ℓ*_1/2_	10	32.756 (2.203)	0.772 (0.031)
DE	44	31.975 (2.701)	0.756 (0.029)
CMA-ES	34.75	32.037 (2.982)	0.736 (0.027)
Elastic net	60	28.721 (3.672)	0.753 (0.022)
*ℓ*_1/2_ + *ℓ*_2_	36	30.333 (2.406)	0.732 (0.026)
CHR-GS	41.667	27.460 (2.181)	0.771 (0.023)
CHR-DE	41	**27.161** (2.026)	**0.781** (0.018)

As see from the Table 6, CHR-DE penalty selects some unique genes, such as KRT14, NOLC1. Liver cytokeratin14 (**KRT14**), a marker of liver stem cells, is only positive in G0 phase of hepatocellular carcinoma cell line Huh7 [44]. **NOLC1** is regulated by CREB-NOLC1 pathway that plays an important role in hepatocellular carcinoma progression by modulating tumor growth, angiogenesis and apoptosis [45, 46]. Furthermore, the ADRB3, MAPK3, MGAT1, TGFBI and DAD1 are selected by CHR-DE penalty and other methods such as Lasso, *ℓ*_1/2_, DE, CMA-ES and CHR-GS meanwhile. Especially, the ADRB3 and MAPK3 in a small group of network by CHR-DE method as shown in Fig 4, and they are also targeted by several cancer drugs. Zhao et al [47] identified two pathways, “calcium signaling pathway” and “neuroactive ligand-receptor interaction” containing **ADRB3**, which correlated with middle and late stages of HCC development. Okabe et al [48] suggested that activation of the MAPK pathway containing **MAPK3, MAPK9** is a common feature of HCC. Guo et al [49] reported alterations of glycogene and N-glycan such as **MGAT1** in human hepatocarcinoma cells correlate with tumor invasion, tumorigenicity and sensitivity to chemotherapeutic drug. As a tumor suppressor, arginylglycylaspartic acid (RGD) peptides released from *β*ig-H3, also known as transforming growth factor-beta-induced protein (**TGFBI**) peptides mediate apoptosis of Hep3B hepatoma cells [50]. While, *β*ig-H3 can promote the progression of hepatocellular carcinoma as well [51, 52]. Tanaka et al [53] has demonstrated that high expression of **DAD1** in HCC cells can activate oligosaccharyltransferase (OST) and block apoptosis, thereby enhancing tumor cell survival.

**Table 6:** The top 10 selected genes in the GSE10141.

	Lasso	*ℓ*_1/2_	DE	CMA-ES	Elastic net	*ℓ*_1/2_ + *ℓ*_2_	CHR-GS	CHR-DE
1	PSG6	CYP24A1	KLRC3	SLC29A2	PSG6	PSG6	CYP24A1	CYP24A1
2	CYP24A1	ADRB3	IFI6	HMGB2	CYP2A7	CYP24A1	ADRB3	KRT14
3	ADRB3	OLFM4	IL32	TTC35	CYP24A1	CYP2A7	ATP6AP2	ADRB3
4	PPP2CA	EFNA5	NCBP2	BTG3	LBX1	GPR3	MGAT1	ATP6AP2
5	MGAT1	MGAT1	ITGA5	ICAM2	SYT5	MPL	SPTBN1	MGAT1
6	CCR9	AADAC	LSR	NFKBIB	SLC10A2	VIP	AUH	TGFBI
7	DAD1	SULT1E1	SPTBN2	MAPK3	KRT81	PRKCQ	IGFBP3	NOLC1
8	ATP6AP2	TGFBI	ASPA	TAP1	MPL	SSTR3	SULT1E1	DAD1
9	CAPZA1	LSR	MAPK9	OSTF1	EPYC	SYT5	GRM5	MAPK3
10	OLFM4	HIST1H2BH	RSC1A1	EIF2B1	HTR6	KRT81	ACTB	GM2A

#### 4.2.3 Colorectal cancer

GSE103479 contains 110,961 genes for 155 colorectal cancer (CRC) patients. Table 7 also shows that the CHR-DE performed best in predicting the patients’ survival time with selecting smaller number of genes than the Elastic net and CHR-GS.

**Table 7:** The results with standard errors in parentheses for GSE103479.

Penalty	# selected genes	MSE	CI
Lasso	39	63.909 (3.588)	0.691 (0.033)
*ℓ*_1/2_	18	62.245 (2.624)	0.725 (0.049)
DE	44	62.374 (3.115)	0.707 (0.034)
CMA-ES	39.5	63.975 (4.415)	0.682 (0.031)
Elastic net	66	61.201 (4.290)	0.713 (0.036)
*ℓ*_1/2_ + *ℓ*_2_	43	59.832 (3.278)	0.727 (0.028)
CHR-GS	56.333	56.202 (3.107)	0.735 (0.036)
CHR-DE	51.333	**53.999** (3.043)	**0.748** (0.035)

As see from the Table 8, the **CDC42** is selected by CHR-DE penalty and other methods. It is one of the best characterized members of the Rho GTPase family, which was found to be up-regulated in several types of human tumors including CRC. Targeting CDC42 would potentially decrease CRC metastasis formation [54, 55, 56]. Furthermore, there are four selected genes CDC42, SLC10A2, TNRC6B and MOV10 in a small group of network by CHR-DE method as shown in Fig 5. This ileal sodium dependent bile acid transporter (ISBT; gene code: **SLC10A2**) has been associated with the risk for development of sporadic colorectal adenoma, a precursor lesion for CRC [57]. **ATN1** may be promising biomarkers for the distinction between serrated and conventional CRC [58]. These two above genes SLC10A2 and ATN1 are selected by CHR-DE penalty and Lasso. The RPS11 is selected by these 6 different penalties at the same time. Kasai et al [59] demonstrated that **RPS11** is highly expressed in CRC (especially in immature mucosal cells located in the crypt base) but can be detected hardly in the normal colorectal mucosa.

**Table 8:** The top 10 selected genes in the GSE103479.

	Lasso	*ℓ*_1/2_	DE	CMA-ES	Elastic net	*ℓ*_1/2_ + *ℓ*_2_	CHR-GS	CHR-DE
1	RPS11	RPS11	TMTC1	CLDND2	RPS11	RPS11	RPS11	RPS11
2	TNRC6B	IK	GALT	ABCD3	FCGR3A	IK	LINC01315	LINC01315
3	LINC01315	RP11-50B3.4	CDC42	KLRK1	FAM24A	RPL27A	FCGR3A	TNRC6B
4	CDC42	RHOA	RMND5B	CDC42	IK	CDC42	IK	SLC10A2
5	SLC10A2	PIAS1	SPACA1	MYH4	RPL27A	TNRC6B	RPL27A	CDC42
6	ATN1	SERPINB12	LILRB1	ITGA7	XKRX	SERPINC1	FAM24A	ATN1
7	SERPINB12	DLST	OR8B2	AVEN	NNMT	GABPA	SERPINB12	MOV10
8	LCE1B	CDC42	OPTC	TBC1D32	DEFB108B	SLC10A2	CDC42	BPIFA3
9	RNF215	RPL27A	GLTSCR2	OR5P3	SERPINB12	XKRX	XKRX	SERPINB12
10	WDR73	ZDHHC20	SYTL1	A4GNT	TREM1	ZDHHC20	TNRC6B	GABPA

## 5 Conclusion

In this paper, we have proposed a penalized accelerated failure time model CHR-DE to recognize the biomarkers that are both biologically meaningful and clinically. This model is designed based on wrapper-embedded memetic framework that combines a non-convex regularization (local search) with differential evolution (global search). First, this new method inherits the robust power of regularization methods that integrate feature selection and learning procedure into a single process. Furthermore, our proposed method utilizes differential evolution (DE) to globally optimize the CHR’s hyperparameters, which make CHR-DE achieve strong capability of selecting groups of genes in high-dimensional biological data. We also developed an efficient path seeking algorithm to optimize this penalized model. The results in both synthetic and real datasets have indicated that the CHR-DE method is highly competitive against some existing feature selection approaches to select biomarkers in groups. Additionally, this CHR-DE scheme can be easily implemented in other high-dimensional and low-sample datasets.

## Supporting information

S1 Appendix The results with different values of MSE and CI weights. We display the results with different weightings in synthetic datasets and breast cancer data (GSE22210).
(PDF)
