# Supplementary material for: Complex harmonic regularization with differential evolution in a memetic framework for biomarker selection
Source: PLoS One. 2019 Feb 14;14(2):e0210786. doi: 10.1371/journal.pone.0210786 (PMC6375558; doi:10.1371/journal.pone.0210786)
Supplement: S1 Appendix — We display the results with different weightings in synthetic datasets and breast cancer data (GSE22210). (PDF) [file pone.0210786.s001.pdf]

S1 Appendix: The results of different values of MSE and CI weights.

We display the experimental settings with different values of mean-squared-error (MSE) and concordance index (CI) weights in synthetic datasets are similar in section 4. In Eq. (13), w_M is the weight of MSE for the individual i in the population, w_C is the CI for this individual and usually $w_M + w_C = 100\%$. These weight factors can be adjusted according to what people value as an important weight. The w_M are 20%, 80%, 100% to represent low, medium and high ratio of importance. As see from the Table 1, $w_M = 80\%,\ w_C = 20\%$ in our proposed CHR-DE approach gives lower MSE with higher CI than other weightings.

Table 1: The results with different values of MSE and CI weights in synthetic datasets. Standard errors are given in parentheses.

$\varrho = 0.2$	Sensitivity	Specificity	MSE	CI
$w_M = 20\%,\ w_C = 80\%$	0.978 (0.042)	0.955 (0.025)	7.166 (1.412)	0.895 (0.014)
$w_M = 100\%,\ w_C = 0$	0.982 (0.064)	0.957 (0.014)	6.656 (1.644)	0.905 (0.016)
$w_M = 80\%,\ w_C = 20\%$	**0.988** (0.081)	**0.962** (0.012)	**6.461** (1.520)	**0.914** (0.012)

We also give the final rank with different values of MSE and CI weights in breast cancer data (GSE22210) in Table 2.

Table 2: The top 10 selected genes with different values of MSE and CI weights in breast cancer data (GSE22210).

	$w_M = 20\%,\ w_C = 80\%$	$w_M = 100\%,\ w_C = 0$	$w_M = 80\%,\ w_C = 20\%$
1	APOA1	SERPINB2	SERPINB2
2	COL1A2	NFKB1	IMPACT
3	PLAU	RUNX3	XIST
4	DBIRC4	ABCA1	HIC1
5	LAT	IGF1R	IGF1R
6	TDGF1	NGF	LAT
7	WEE1	DSG1	LIF
8	EFZD9	FGF12	IL1B
9	GJB2	ASB4	NFKB1
10	SERPINB2	EPS8	RASA1
